# Diagnóstico Eletrocardiográfico da Hipertrofia Ventricular Esquerda

**DOI:** 10.36660/abc.20210868

**Published:** 2021-11-01

**Affiliations:** 1 Universidade Federal do Paraná - Cardiologia Curitiba PR Brasil Universidade Federal do Paraná - Cardiologia, Curitiba, PR – Brasil

**Keywords:** Hipertrofia Ventricular Esquerda, Diagnóstico por Imagem, Eletrocardiografia/métodos, Ecocardiografia/métodos, Cardiomiopatia Hipertrófica, Disfunção do Ventrículo Esquerdo

A Hipertrofia Ventricular Esquerda (HVE) é definida como um aumento da massa do ventrículo esquerdo (VE), que pode ser secundária a um aumento da espessura das paredes (HVE concêntrica), aumento do tamanho da cavidade (HVE excêntrica) ou ambos. A apresentação do VE hipertrofiado depende principalmente da doença subjacente, sendo a HVE concêntrica resultante na maioria dos casos da sobrecarga de pressão no VE (hipertensão arterial ou estenose aórtica), enquanto a HVE excêntrica depende principalmente das sobrecargas de volume no VE (insuficiências mitral e aórtica) e cardiomiopatias dilatadas. Outras causas de HVE incluem os defeitos do septo ventricular, cardiomiopatia hipertrófica e alterações fisiológicas associadas ao treinamento atlético.^1^

A presença de HVE é clinicamente importante por estar associada com aumento da incidência de insuficiência cardíaca, arritmias ventriculares, insuficiência vascular periférica, dilatação da aorta, eventos cerebrovasculares e morte súbita ou após infarto do miocárdio.^2^

A HVE pode ser diagnosticada pelo eletrocardiograma (ECG) ou pelo ecocardiograma, sendo este o procedimento de escolha por ter sensibilidade muito maior que o ECG.^3^ O ECG é uma ferramenta útil, mas imperfeita na detecção da HVE; sua utilidade se deve principalmente ao baixo custo e sua disponibilidade universal, sendo realizado rotineiramente nas avaliações cardiológicas. O ecocardiograma tem um custo maior, mas não exagerado, e também tem estado amplamente disponível. Ainda, para avaliação da massa ventricular são empregadas as técnicas mais acessíveis do método. Em poucas situações a ressonância magnética cardíaca pode ser necessária, só quando as condições técnicas inviabilizem a avaliação ecocardiográfica.^4^

O cálculo da massa ventricular esquerda pela ecocardiografia pode ser feito por diferentes técnicas – unidimensional, bidimensional ou tridimensional, mas sempre com o objetivo de quantificar o miocárdio daquela câmara, baseado em fundamentos comuns e, portanto, com resultados semelhantes. Os padrões de normalidade são preconizados pelas associações internacionais de ecocardiografia (ASE, EACI)^5^ e endossados pela maioria dos autores.^6^ Desta forma se observa na ecocardiografia uma uniformidade dos resultados da HVE, baseados em poucos parâmetros estudados.^5,6^

Na eletrocardiografia a situação é oposta. Já em 1969, Romhilt et al.^7^ descreviam 33 critérios eletrocardiográficos para o diagnóstico de HVE, e todos se mostravam com baixa sensibilidade.^7^ No correr dos anos, alguns critérios se solidificaram como os mais empregados na prática clínica para o diagnóstico da HVE no ECG, mas ainda não há consenso nesta seleção. Em artigo recente, Wang et al.,^8^ estudaram o desempenho de sete critérios do ECG em pacientes chineses com HVE no ecocardiograma. Encontraram uma sensibilidade de 15%-31,9% e especificidade de 91,6%-99,2% na amostra global, com melhor sensibilidade na HVE concêntrica. Os melhores descritores de HVE nesta pesquisa^8^ foram os critérios de Sokolow-Lyon voltagem, Cornell voltagem, Cornell produto e R aVL voltagem.

Povoa et al.,^9^ em publicação nesta revista, estudaram 13 critérios eletrocardiográficos de HVE em 2458 pacientes hipertensos submetidos a ecocardiograma, classificados pela faixa etária e submetidos a rigorosa análise estatística. Entre os pacientes com idade ≥ 80 anos tiveram melhor desempenho os critérios de Perugia (sensibilidade 44,7%, especificidade 89,3% e DOR - *diagnostic odds ratio:* 6,8) e (Rmax + Smax) x duração (sensibilidade 39,4%, especificidade 91,3%, DOR 6,8). Nos pacientes com idade < 80 anos, além destes índices citados, o critério de Narita, descrito em 2019,^10^ também teve um bom desempenho. Nesta pesquisa, tradicionais índices tiveram sensibilidade diagnóstica inferior: Sokolow-Lyon voltagem > 35 mm com 12%-15,7% nas diferentes faixas etárias e Cornell voltagem com 17,3%-21% de sensibilidade.^9^

Entendemos, em conclusão, que o eletrocardiograma continua sendo importante ferramenta na prática cardiológica diária, bastante valioso quando indica HVE, mas com sensibilidade diagnóstica ainda modesta, apesar das novas pesquisas nesta área.
