# Supplementary material for: Diagnóstico Eletrocardiográfico da Hipertrofia Ventricular Esquerda
Source: Arq Bras Cardiol. 2021 Nov 1;117(5):932–3. [Article in Portuguese] doi: 10.36660/abc.20210868 (PMC8682103; doi:10.36660/abc.20210868)
Supplement: Translation

# Translation: Electrocardiographic Diagnosis of Left Ventricular Hypertrophy

**Affiliations:** 1 Curitiba PR Brazil Universidade Federal do Paraná - Cardiologia, Curitiba, PR – Brazil

**Keywords:** Hypertrophy, Left Ventriculat, Diagnostic Imaging, Electrocardiography/methods, Echocardiography/methods, Cardiomyopathy, Hypertrophic, Ventricular Dysfunction, Left

Left Ventricular Hypertrophy (LVH) is defined as an increase in left ventricular (LV) mass, which may be secondary to an increase in wall thickness (concentric LVH), increased cavity size (eccentric LVH), or both. The presentation of hypertrophied LV depends mainly on the underlying disease, with concentric LVH resulting in most cases from LV pressure overload (hypertension or aortic stenosis), while eccentric LVH mainly depends on LV volume overloads (mitral and aortic insufficiency) and dilated cardiomyopathies. Other causes of LVH include ventricular septal defects, hypertrophic cardiomyopathy, and physiological changes associated with athletic training.^1^

The presence of LVH is clinically meaningful because it is associated with an increased incidence of heart failure, ventricular arrhythmias, peripheral vascular insufficiency, aortic dilatation, cerebrovascular events and sudden death or after myocardial infarction.^2^

LVH can be diagnosed by electrocardiogram (ECG) or echocardiogram, which is the procedure of choice because it has a much greater sensitivity than the ECG.^3^ The ECG is a useful but imperfect tool in detecting LVH; its usefulness is mainly due to its low cost and universal availability, routinely performed in cardiac evaluations. Echocardiography is more expensive but not unreasonable and has also been widely available. Yet, to assess the ventricular mass, the most accessible techniques of the method are used. In few situations, cardiac magnetic resonance imaging may be necessary, only when technical conditions make echocardiographic assessment unfeasible.^4^

The calculation of left ventricular mass by echocardiography can be performed using different techniques – one-dimensional, two-dimensional or three-dimensional, but always to quantify the myocardium in that chamber, based on common fundamentals and, therefore, with similar results. Standards of normality are recommended by the international associations of echocardiography (ASE, EACI)^5^ and endorsed by most authors.^6^ Thus, echocardiography shows uniformity of LVH results based on few studied parameters.^5,6^

In electrocardiography, the situation is the opposite. As early as 1969, Romhilt et al.^7^ described 33 electrocardiographic criteria for diagnosing LVH, and all showed low sensitivity.^7^ Over the years, some criteria have solidified as the most used in clinical practice for diagnosing LVH on the ECG, but there is still no consensus in this selection. In a recent article, Wang et al.^8^ studied the performance of seven ECG criteria in Chinese patients with LVH on echocardiography. They found a sensitivity of 15%-31.9% and a specificity of 91.6%-99.2% in the global sample, with better sensitivity in concentric LVH. The best LVH descriptors in this research^8^ were the Sokolow-Lyon voltage, Cornell voltage, Cornell product and R aVL voltage criteria.

Povoa et al.,^9^ in a publication in this journal, studied 13 electrocardiographic criteria for LVH in 2458 hypertensive patients submitted to echocardiography, classified by age group and submitted to rigorous statistical analysis. Among patients aged ≥ 80 years, the Perugia criteria performed better (sensitivity 44.7%, specificity 89.3% and DOR - diagnostic odds ratio: 6.8) and (Rmax + Smax) x duration (sensitivity 39.4 %, specificity 91.3%, DOR 6.8). In patients aged < 80 years, in addition to these indices mentioned above, the Narita criterion, described in 2019,^10^ also performed well. In this research, traditional indices had lower diagnostic sensitivity: Sokolow-Lyon voltage > 35 mm with 12%-15.7% in different age groups and Cornell voltage with 17.3%-21% sensitivity.^9^

In conclusion, we understand that the electrocardiogram remains an important tool in daily cardiology practice, quite valuable when it indicates LVH, but with still modest diagnostic sensitivity, despite new research in this area.
